# HIV-1 Subtype distribution in morocco based on national sentinel surveillance data 2004-2005

**DOI:** 10.1186/1742-6405-9-5

**Published:** 2012-02-14

**Authors:** Mohammed Akrim, Sanae Lemrabet, Elmir Elharti, Rebecca R Gray, Jean Claude Tardy, Robert L Cook, Marco Salemi, Patrice Andre, Taj Azarian, Rajae El Aouad

**Affiliations:** 1 Molecular Biology Unit, National Reference Laboratory for HIV, National Institute of Hygiene, Rabat, Morocco; 2 HIV Diagnosis and Immunologic Follow up Unit, National Reference Laboratory for HIV, National Institute of Hygiene, Rabat, Morocco; 3 Department of Zoology, University of Oxford, Oxford, UK; 4 Laboratoire de Virologie, Hôpital de la Croix Rousse, Lyon, France; 5 Department of Epidemiology and Emerging Pathogens Institute, University of Florida, Gainesville, USA; 6 Department of Pathology, Immunology and Laboratory Medicine, School of Medicine and Emerging Pathogens Institute, University of Florida, Gainesville, USA

**Keywords:** HIV-1, subtypes, phylogeny, Morocco

## Abstract

**Background:**

Little is known about HIV-1 subtype distribution in Morocco. Some data suggest an emergence of new HIV subtypes. We conducted phylogenetic analysis on a nationally representative sample of 60 HIV-1 viral specimens collected during 2004-2005 through the Morocco national HIV sentinel surveillance survey.

**Results:**

While subtype B is still the most prevalent, 23.3% of samples represented non-B subtypes, the majority of which were classified as CRF02_AG (15%). Molecular clock analysis confirmed that the initial introduction of HIV-1B in Morocco probably came from Europe in the early 1980s. In contrast, the CRF02_AG strain appeared to be introduced from sub-Saharan Africa in two separate events in the 1990s.

**Conclusions:**

Subtype CRF02_AG has been emerging in Morocco since the 1990s. More information about the factors introducing HIV subtype-specific transmission will inform the prevention strategy in the region.

## Introduction

HIV-1 variability remains a formidable challenge for designing a protective vaccine or an effective cure. The HIV-1 is divided into 4 groups: M, N, O and P. Group M is responsible for the current pandemic and includes more than 49 circulating recombinant forms (CRFs), 9 subtypes, 5 sub-subtypes, and unique recombinant forms (URFs) [1,2]. HIV genetic diversity is generated by the high rate of virus mutation, rapid viral turnover and frequent recombination events between subtypes [3]. Furthermore, there is an unequal geographic distribution of HIV-1 subtypes and CRFs around the world characterized by different epidemic behaviours and growth rates [4]. For instance, in western Europe and North America, subtype B is the most prevalent whereas in sub-Saharan Africa subtypes A, C, D and CRF02_AG predominate [5-7]. This geographic distribution of HIV-1 subtypes could result from migration, travel, or geographic accessibility. These factors may contribute to the transmission of these clades outside the regions where they are most prevalent [8,9]. The increasing diversity of HIV-1 underscores the need for diagnostics, patient monitoring tools, and treatment options that are effective across the full spectrum of known groups, subtypes, and recombinant forms.

The first reported case of HIV/AIDS in Morocco occurred in 1986. Up to December 2010, a cumulative total of 2,914 persons have been diagnosed with AIDS in Morocco, and estimates suggest approximately 26,000 persons are living with HIV in the country [10]. Among them, 58% were identified during the 6 last years. Furthermore, more than half of cases are from 3 regions: the Agadir region (22%), the Marrakech region (16%) and the Casablanca region (14%). Young adults (15-39 years) represent 64% of all the cases, and the proportion of HIV infections in women has increased from 18% (1986-1990) to more than 40% (2004-2008). HIV-1 transmission is reportedly attributed to heterosexual transmission in more than 80% of individuals.

A national HIV sentinel surveillance network has been implemented in Morocco since 1993 [11]. This surveillance is based on an anonymous, unlinked study and is approved by the WHO Ethical Committee. Studied groups include pregnant women, patients consulting healthcare centres with STIs, persons with tuberculosis, prisoners, injecting drug users (IDUs) and sex workers.

A study of HIV subtypes in Morocco from 1997 showed a predominance of HIV-1 subtype B (93.5%), a pattern more similar to Europe than sub-Saharan Africa [12]. More recently, an analysis of HIV subtypes from a single region of Morocco suggests an increase in persons with HIV CRF02-AG, which has typically been associated with infections from sub-Saharan Africa [13]. Since the mid-1990s, Morocco has been experiencing a significant immigration of persons from sub-Saharan Africa, many of which are attempting to enter Europe. More recently, Spain and the European Union have intensified their border and coastal surveillance. Consequently, Morocco has shifted from being a transit country to the final station for many migrants. Other countries in the MENA region are also demonstrating a more diverse HIV epidemic in terms of HIV subtype distribution [14].

The study of HIV subtype distribution may reveal epidemiological patterns of transmission or distinct networks associated with specific risk behaviours. Phylogenetic analysis can further expand the identification of specific epidemiological clusters of HIV infection from a common origin [9]. We investigated the pattern of HIV-1 subtype diversity and high-resolution phylogenetic analysis of a representative sample of 60 HIV-infected persons identified through the Morocco national HIV sentinel surveillance program.

## Materials and methods

### Sample collection

Samples were collected as part of the sentinel surveillance system, a national HIV epidemic AIDS surveillance survey carried out each spring by the Moroccan Ministry of Health to assess trends in the HIV epidemic. The sera were collected from different regions of Morocco during 2004 and 2005. All of the 60 HIV-positive samples during the 2004-2005 survey were included in this study. Samples were screened HIV-1 positive by Elisa (HIV1/2 Genscreen plus, Bio-Rad, France) and confirmed by Western blot test (Genlabs, USA). These samples were representative of the different regions of Morocco and were consistent with the age and sex distribution of reported HIV cases within the country (Table 1).

**Table 1:** Database of HIV sequences included in the study

Sample Code	N°	Origin	Risk Group	Sex	Age	Sub-type PR	Subtype RT
N1BBB	B	Tetouan	Patient with TB	M	35	B	B

N1CBB	C	Tetouan	Patients with STI's	M	32	B	B

N1A B	A	Tetouan	Patient with TB	M	39		B

N2NBB	N	Rabat	Male prisoners	M	30	B	B

N238B	38	Rabat	Female SW in prison	F	19	B	

S4DBB	D	Casablanca	Patients with STI's	F	41	B	B

S4EBB	E	Casablanca	Patient with TB	M	39	B	B

S4Q1BB	Q1	Casablanca	Pregnant women	F	19	B	B

S3MBB	M	Beni-Mellal	Male prisoners	M	25	B	B

S3LBB	L	Safi	NA			B	B

S2J1BB	J1	Marrakech	Male prisoners	M	28	B	B

S136BB	36	Agadir	Patients with STI's	F	35	B	B

S1B1BB	B1	Agadir	Female SW in MC	F	28	B	B

S1C1BB	C1	Agadir	Patients with STI's	F	30	B	B

S1D1BB	D1	Agadir	Marines	M	28	B	B

S1E1BB	E1	Agadir	Pregnant women	F	38	B	B

S1F1BB	F1	Agadir	Female SW in prison	F	21	B	B

S1G1BB	G1	Agadir	Female SW in prison	F	21	B	B

S1RBB	R	Agadir	Patient with TB	M	20	B	B

S1VBB	V	Agadir	Male prisoners	M	24	B	B

S1ZBB	Z	Agadir	Patients with STI's	F	18	B	B

S1V1BB	V1	Taroudant	Pregnant women	F	38	B	B

S235BB	35	Marrakech	Patients with STI's	M	39	B	B

SX2K1O1BB		Marrakech	NA			B	B

S2I1BB	I1	Marrakech	Patients with STI's	F	23	B	B

S2L1BB	L1	Marrakech	Pregnant women	F	27	B	B

S2M1BB	M1	Marrakech	Male prisoners	M	25	B	B

S2N1BB	N1	Marrakech	Male prisoners	M	22	B	B

S1WBB	W	Agadir	Female SW in prison	F	25	B	B

S115DB	15	Agadir	Male prisoners	M	25	B	B

S1A1DB	A1	Agadir	Male prisoners	M	27	B	B

S114DB	14	Oulad Taima	Female SW in MC	F	31	B	B

S118DB	18	Oulad Taima	Pregnant women	F	30	B	B

S3JBB	J	Safi	Male prisoners	M	28	B	B

S29BB	9	Marrakech	Patients with STI's	F	22	B	B

S216BB	16	Marrakech	Patients with STI's	M	40	B	B

SX1K1O1BB		Agadir	NA	29	29	B	B

S1H1BB	H1	Agadir	Pregnant women	F	24	B	B

S225B	25	Marrakech	Male prisoners	M	29	B	

S240B	40	Chichaoua	Patients with STI's	F	34	B	

S28B	8	Chichaoua	Patients with STI's	F	44	B	

S311B	11	Safi	Pregnant women	F	26	B	

S3KB	K	Safi	Patients with STI's	F	41	B	

S139B	39	Agadir	Patients with STI's	F	25	B	

S13B	3	Oulad Taima	Female SW in MC	F	29	B	

S113 B	13	Oulad Taima	Female SW in MC	F	39		B

N319CC	19	Meknes	Patients with STI's	F	21	C	C

N124AC	24	Tanger	Drug users	M	27	A	C

N220BC	20	Rabat	Patient with TB	F	33	B	C

N241AAE	41	Rabat	Patients with STI's	F	25	A	AE

N3HAGAG	H	Meknes	Pregnant women	F	22	AG	AG

N229AGAG	29	Rabat	Patients with STI's	F	26	AG	AG

N2QAGAG	Q	Rabat	Patients with STI's	F	29	AG	AG

N2OAG	O	Rabat	Patients with STI's	F	29	AG	

N2PAG	P	Rabat	Patients with STI's	M	28	AG	

N446AG	46	Oujda	Patients with STI's	F	40	AG	

S1TAGAG	T	Agadir	Hotels workers	M	35	AG	AG

S1SAGB	S	Agadir	Female SW in MC	F	30	AG	B

S27AGAG	7	Chichaoua	Consultant for STI	F	24	AG	AG

S317AGAG	17	Safi	Consultant for STI	F	39	AG	AG

### PCR and DNA sequencing

To study diversity, samples were sequenced in *pol *gene region [protease gene (PR) and 2/3 5' region of the reverse transcriptase gene (RT)]. The viral RNA was extracted and PR and RT genes were RT-PCR amplified as previously described [15]. The fragments obtained were sequenced on both strands using an automated sequencer Beckman CEQ 2000 DNA Analysis System, and subtyped by using the Rega HIV-1 Subtyping tool version 2.0 (http://dbpartners.stanford.edu/RegaSubtyping).

GenBank accession numbers for the sequences reported in this study are JQ316543 to JQ316600 and JQ344156 to JQ344204 for PR and RT sequences respectively.

### Dataset assembling

All sequences from Morocco were divided into protease (PR, N = 58) and reverse transcriptase (RT, N = 49) alignments (Table 1). A set of full genome reference sequences (Genbank:AF004885, Genbank:AB253421, Genbank:AB253429, Genbank:AF286241, Genbank:AF286237, Genbank:K03455, Genbank:AY423387, Genbank:AY173951, Genbank:AY331295, Genbank:DQ853463, Genbank:U52953, Genbank:U46016, Genbank:AF067155, Genbank:AY772699, Genbank:K03454, Genbank:AY371157, Genbank:AY253311, Genbank:U88824, Genbank:AF077336, Genbank:AF005494, Genbank:AF075703, Genbank:AJ249238, Genbank:AY371158, Genbank:AJ249236, Genbank:AJ249237, Genbank:AF377956, Genbank:AF084936, Genbank:AF061641, Genbank:U88826, Genbank:AF190127, Genbank:AF190128, Genbank:AF005496, Genbank:EF614151, Genbank:AF082394, Genbank:AF082395, Genbank:AJ249235, Genbank:AJ249239, Genbank:AY271690) were downloaded from the Los Alamos HIV database. An additional set of sequences of PR/RT for subtypes HIV-1 CRF02-AG and subtype B were downloaded from the Los Alamos database. The criterion for inclusion in this dataset included the following: sequences were published, were not amplified in culture and had a known location and year of sampling. The HIV-1B dataset was reduced for computational practicality and included representative sequences only from major geographic areas. Multiple-sequence alignments were obtained by codon-alignment with the CLUSTAL algorithm, and subsequently manually edited for optimization (Alignments are available from the authors upon request).

### Phylogenetic analysis

Maximum likelihood (ML) trees were first inferred using the Moroccan sequences and full genome references sequences. Analyses were performed assuming the GTR + Gamma model of nucleotide evolution. Statistical support was assessed by non-parametric bootstrapping (number of replicates = 500) using PHYML version 3.0 [16]. Sequences that clustered with a pure subtype with a bootstrap value of > 80 were classified as such. Sequences that clustered with the CRF02_AG with a bootstrap value > 50 were classified as HIV-1 CFR02_AG. All Moroccan sequences with a confirmed subtype were assembled, and sequences from the same subject with concordant subtypes in PR and RT were concatenated. MLtrees were inferred using the final alignments for each subtype using concatenated PR/RT sequences. Analyses were performed assuming the GTR + Gamma model of nucleotide evolution. Statistical support was assessed by non-parametric bootstrapping (number of replicates = 500) using PHYML.

#### Molecular clock analysis

The evolutionary rate (nucleotide substitutions per site per year) and the time of the most recent common ancestor (T_MRCA_, years) of HIV-1B in Morocco were inferred using sequences sampled at different time points by the MCMC approach implemented in BEAST [17]. The analyses were performed with the same nucleotide substitution model described in the previous section, and different coalescent priors (constant, exponential and Bayesian Skyline Plot), assuming a strict or a relaxed molecular clock [18]. An MCMC was run for 100,000,000 generations with sampling every 10,000^th ^generation. The results were visualized in Tracer. The effective sample size (ESS) value for each parameter was > 500 indicating sufficient mixing of the Markov chain.

## Results

Sixty HIV-1 positive sera were genotyped: 41 from the south (68.3%), 14 (23.3%) from the center and 5 (8.3%) from the North of Morocco. Sex distribution was 62% females and 38% males, with an average age of 29 years. The risk groups represented in the study sample included persons attending STI clinics (38.6%), male prisoners (17.5%), female sex workers (15.8%), pregnant women (14%), people with TB (8.8%), drug users (5.3%), marines (1.7%) and hotel workers (1.7%) (Table 1).

The PR and RT sequences were both positive for 47 (78.3%) samples. Of these, 43 (91.5%) had concordant subtype assignments including 36 (83.7%) subtype B, 6 (14%) CRF02_AG and 1 (2.3%) subtype C (Table 1). The remaining 4 (8.5%) samples in which both PR and RT regions were positive revealed discordant subtypes that represent intersubtypes and/or inter-CRF recombinant viruses. They include B/C, A/C, A/CRF01_AE and CRF02_AG/B which are represented by one sample each. Finally, of the 13 specimens with HIV-1 subtype assignment for only one viral region, 8 PR and 2 RT sequences were of subtype B and 3 PR sequences were of subtype CRF02-AG.

HIV-1 subtypes appeared to be differently distributed in Moroccan geographic regions. Subtype B strains appeared to be widely distributed with little geographic compartmentalization from region to region, whereas the single samples of subtype C, A/C, B/C and A/CRF01_AE were all concentrated in the northern regions of Morocco.

**Figure 1:**
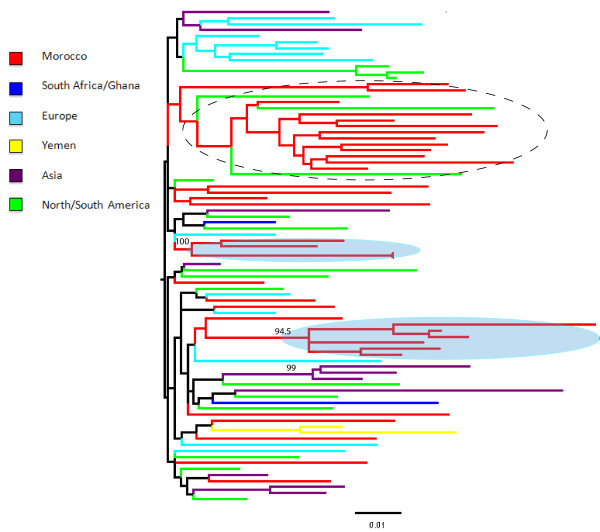
**HIV-1B Maximum likelihood (ML) trees**. The ML tree includes HIV-1B Moroccan sequences for which we have RT and PR sequences, as well as 46 subtype B reference sequences from the HIV database that were randomly chosen to represent major geographic areas in the world. The tree was generated using the GTR+G model of nucleotide substitution using the concatenated *RT *and *PR *genes. Branches are drawn in scale, according to the bar at the bottom, and colored to reflect geographic origin according to the legend of the figure. The number along a branch indicates significant bootstrap support (> 65%). Sequences were named using the year of sampling preceded by the two letter county code of origin, according to the HIV database guidelines.

Figure 1 shows a ML tree, including HIV-1 subtype B sequences from Morocco as well as worldwide reference sequences downloaded from the HIV databases (http://www.hiv.lanl.gov/content/index). Overall, Moroccan strains are highly intermixed with reference strains from different geographic regions, suggesting multiple introductions of subtype B in Morocco over a relatively long period of time. Two highly supported monophyletic clades (100% and 94.5% bootstrap support, respectively) of Moroccan strains appear to be related to HIV-1B sequences from Europe, whereas a third large clade clustered together with sequences from the United States, although the clade was only weakly supported by bootstrapping (< 50%). The time of the most recent common ancestor (TMRCA) of HIV-1B Moroccan strains calculated by molecular clock analysis dated back to 1983 (95% high posterior density intervals: 1975-1987) according to the constant population size coalescent prior enforcing a relaxed molecular clock. Different coalescence priors also produced very similar estimates (data not shown).

**Figure 2:**
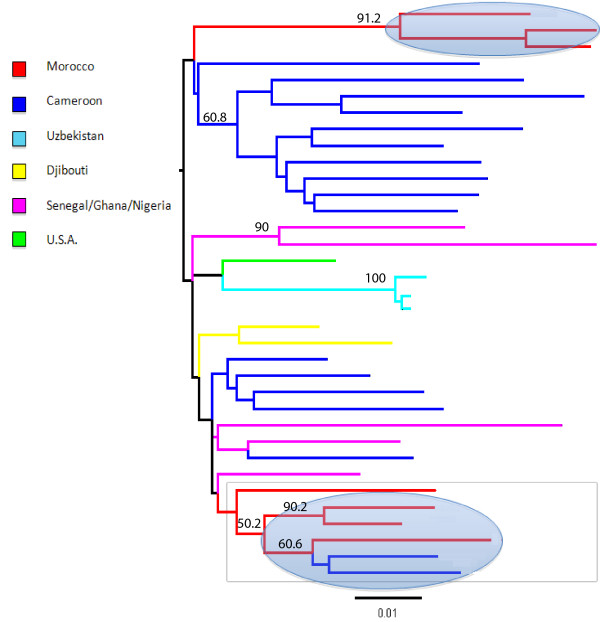
**HIV-1 CRF02_AG Maximum likelihood (ML) trees**. The ML tree includes HIV-1 Moroccan CRF02_AG sequences, for which we have RT and PR sequences, together with 28 CRF02_AG strains downloaded from the HIV databases for which the full genome sequences were available. The tree was generated using the GTR+G model of nucleotide substitution using the concatenated *RT *and *PR *genes. Branches are drawn in scale, according to the bar at the bottom, and colored to reflect geographic origin according to the legend of the figure. The number along a branch indicates significant bootstrap support (> 65%). Sequences were named using the year of sampling preceded by the two letter county code of origin, according to the HIV database guidelines.

Figure 2 shows a ML tree of Moroccan and reference CRF02_AG strains available in HIV databases. In contrast to the subtype B ML tree, the Moroccan strains are highly localized in two distinct monophyletic clades related to sequences from Cameroon and Senegal. Although the result should be interpreted with caution, given the relatively small number of available sequences for phylogenetic comparison, the tree suggests two separate introductions of CRF02_AG in Morocco from sub-Saharan Africa, dated in 1995 and 1998 respectively, according to the constant population size coalescent prior enforcing a relaxed molecular clock. Again, different coalescence priors had little effect on the estimates (data not shown).

## Discussion

We present the first data on the molecular epidemiology of HIV-1 in Morocco from the national HIV sentinel surveillance survey data. As of 2005, subtype B is still predominant (76.7%), yet following subtype B, there is a high diversity of non-B subtypes, especially CRF02_AG recombinant (15%). Geographic subtype repartition suggests the co-evolution of a more ancient diffusion of European subtype B, and of a more recent spread of sub-Saharan African strains in some Moroccan regions.

These results demonstrate a high diversity of HIV-1 strains in Morocco. This is different from what was reported in 1997 where the distribution of subtype B, A and F strains in Morocco were 93.5%, 1.0%, and 0.5% respectively [12]. However, these results are consistent with our previous results [19] and the more recent results described from the Casablanca region [13]. These findings are also consistent with results described in other countries of the region, including the neighbouring West African countries [14]. The increase of HIV non-B subtypes was also recently reported in many Western Europe countries. Studies conducted in France, Spain, Switzerland, and Portugal have found that the proportion of non-B subtypes may exceed 20% [20,21]. CRF02_AG, which predominates in West Africa, is increasingly more prevalent among the non-B subtypes in these Western European countries.

The overall incidence of HIV-1 in Morocco has been increasing at approximately 15% per year since 2000. The increasing incidence of HIV combined with the identification of additional non-B subtypes raises concerns regarding the control of the current epidemic. The entry of new HIV recombinant viruses is likely the consequence of active exchange between different populations, such as Moroccan groups at risk and persons migrating through Morocco from sub-Saharan Africa. Before 1997, the presence of Sub-Saharan African individuals in Morocco was mostly limited to students and tourists. However, migration of people from sub-Saharan Africa to and through Morocco has been increasing since the late 1990s. In 2007, the Moroccan Ministry of the Interior estimated that approximately 15,000 irregular migrants flow through Morocco each year [22]. In response, the European Union has tightened its boarder control and immigration policies. As a result, many of the migrants settle in Morocco, waiting for an opportunity to cross into Europe. In addition, regular and irregular migrants face many economic and social issues that may increase their risk for HIV transmission. For example, issues such as human trafficking and prostitution could contribute to the circulation of non-B HIV subtypes such as CRF02_AG throughout the country.

The fact that subtype B was more distributed throughout the country, especially in the big-touristic cities (Agadir, Marrakech and Casablanca), suggest this subtype may reflect an older infection. Persons with subtype CRF02_AG were also widely distributed geographically; however, this subtype was not detected in Morocco before 1997, suggesting a more recent epidemic. These findings are also supported by our molecular clock analysis. The other non-B subtypes and recombinants represented more localised transmission due to C, A/C, B/C and A/CRF01_AE strains in the northern part of Morocco.

As the first case of HIV/AIDS in Morocco was reported in 1986, there is an excellent agreement with the TMRCA of HIV-1B of 1983 estimated by molecular clock analysis. Since that date, HIV has been spreading throughout the country, mainly by heterosexual transmission [11]. According to the sentinel surveillance system, the overall HIV prevalence is less than 1% in Morocco. However, even though Morocco is a low prevalence epidemic, HIV/AIDS cases are steadily rising, chiefly in the southern Morocco region of Agadir and neighbouring areas that may represent the epicentre of the epidemic within Morocco [23].

Our findings should be interpreted in light of study limitations. While our analysis included all HIV positive specimens from the 2004-2005 survey, the sample size was relatively small. Therefore, it is not possible to generalise the results as a national trend in Morocco. In addition, routes of transmission and clinical and immunologic status of the HIV-infected individuals were not available for this study, since they are not required in the surveillance process. However, the present data should prompt us to continue to track the molecular epidemiology of the HIV virus in Morocco at the national level. In this context, reinforcement of preventive measures to limit the spread of the epidemic is crucial. Lastly, by limiting our phylogenetic analysis to only the *pol *gene region, we may have missed some recombinants and therefore underestimated their distribution. However, our main finding that CRF02_AG is increasing in Morocco, signifying a shift from an epidemic previously dominated by serogroup B, remains true. In conclusion, the results of this study displayed that HIV diversity is more dynamic in Morocco and its pattern is shifting from the European to sub-Saharan one, i.e. with more subtypes non-B, namely the CRF02_AG. However, more studies to confirm the trend observed during this study and to better characterize the molecular HIV epidemic in Morocco will be of great importance. When taken together, these data demonstrate a dynamic evolution in the HIV diversity in Morocco. The emergence of new HIV subtypes are characterised by an important presence of non-B subtypes that appear to be linked to sub-Saharan populations. More data are needed to better understand the factors responsible for the introduction and spread of new HIV-1 subtype epidemics into regions where they did not exist previously.

## Abbreviations

**AIDS**: Acquired Immune Deficiency Syndrome; **CRF**: circulating recombinant factor; **ESS**: effective sample size; **HIV-1**: Human Immunodeficiency Virus 1; **IDU**: injection drug users; **MENA: **Middle East and North Africa; **ML**: Maximum Likelihood; **PCR**: polymerase chain reaction; **PR**: Protease; **RT**: Reverse Transcriptase; **STI**: Sexually Transmitted Infections; **TB**: tuberculosis; **TMRCA**: Time to Most Recent Ancestor; **WHO**: World Health Organization

## Competing interests

The authors declare that they have no competing interests.

## Authors' contributions

MA: Conduction of the study, samples collection, sequencing and analysis, preparation of the manuscript; SL: sample preparation, sequencing and analysis; EE: sample diagnosis and collection, HIV screening and confirmation, preparation of the manuscript; RG: phylogenetic analysis of samples and construction of phylogenetic trees; JCT: sequencing and analysis, preparation of the manuscript; RLC: interpretation of epidemiological data, preparation of the manuscript; MS: phylogenetic analysis of samples and construction of phylogenetic trees, preparation of the manuscript; PA: sequencing and analysis, preparation of the manuscript; TA: preparation of the manuscript; REA: conduction of the study, preparation of the manuscript. All authors have read and approved the final manuscript.
